# Towards enhancing photostability and adeno-associated viral vector delivery for genetically encoded plasma fluorescent labels

**DOI:** 10.1117/1.NPh.12.S2.S22811

**Published:** 2025-12-12

**Authors:** Philip Gade Knak, Marta Vittani, Ashley Bomin Lee, Laura Nedergaard, Nathalie Vikkelsø Elleholm, Xiaowen Wang, Zuzanna Bojarowska, Maria Celine Martens, Ayumu Konno, Hirokazu Hirai, Maiken Nedergaard, Hajime Hirase

**Affiliations:** a University of Copenhagen, Faculty of Health and Medical Sciences, Center for Translational Neuromedicine, Copenhagen, Denmark; b University of Southern Denmark, Institute of Molecular Medicine—Neurobiology Research, Odense, Denmark; c Gunma University Graduate School of Medicine, Department of Neurophysiology & Neural Repair, Maebashi, Japan; d Gunma University, Initiative for Advanced Research, Viral Vector Core, Maebashi, Japan; e University of Rochester Medical Center, Center for Translational Neuromedicine, Rochester, New York, United States

**Keywords:** microcirculation imaging, cerebral blood flow, adeno-associated virus, fluorescent protein, StayGold

## Abstract

**Significance:**

Investigating blood microcirculation is important to better understand cardiovascular, metabolic, and neurodegenerative diseases. Although visualization of microcirculation has traditionally relied on the injection of short-lived fluorescent tracers, this approach poses challenges for experiments in awake animals or long-term imaging. To address these limitations, we previously developed a genetically encoded fluorescent plasma label that enables stable, minimally invasive *in vivo* visualization of vascular dynamics. Further optimizing its versatility and delivery will expand its utility in neuroscience and across diverse areas of biomedical research.

**Aim:**

We aim to extend the utility of genetically encoded plasma labeling by testing brighter, more photostable fluorescent proteins and evaluating strategies to enhance adeno-associated viral (AAV)-mediated expression.

**Approach:**

AAV8 vectors encoding albumin fused to mNeonGreen or StayGold variants were administered systemically. Expression levels were tracked via blood sampling, and the effects of secondary AAV injection and immunomodulation were tested.

**Results:**

Alb-mStayGold showed improved photostability but lower brightness than Alb-mNeonGreen. Secondary intraperitoneal AAV delivery successfully induced transgene expression even after prior AAV exposure. Moreover, higher AAV8-induced plasma label expression in males is confirmed. Immunosuppressant treatment increased plasma fluorescence, whereas neonatal AAV injection failed to induce tolerance.

**Conclusion:**

Photostable plasma labeling and immune modulation strategies expand the applicability of AAV-based vascular imaging.

## 1 Introduction

Blood microcirculation is a fundamental mechanism for supplying nutrients and exchanging respiratory gases in our organs and tissues, including the brain. For basic research, visualization of microcirculation has been achieved by injecting fluorescent tracers via intravenous routes, such as tail vein injection of fluorescein isothiocyanate dextran.^1^ Although this procedure is well suited for *in vivo* imaging of microcirculation dynamics in acute experiments under anesthetized conditions, its application in experiments with unanesthetized mice or long-term imaging is limited due to the physical nature of the injection route and the short in-blood lifetime of the fluorescent reagent (1 to 2 h). Consequently, repeated injections are needed, which can exert stress on the subject. To overcome these limitations, we have engineered a genetic approach to label blood plasma by recombinant albumin tagged with a fluorescent protein.^2^^,^^3^

Albumin is the most abundant blood plasma protein, present at a concentration of ≃450 micromolar. Plasma albumin is produced by hepatocytes in the liver and secreted into the bloodstream therein. We constructed an adeno-associated viral (AAV) vector that expresses mNeonGreen-fused albumin (Alb-mNG) under the strong hepatocyte-selective P3 transthyretin promoter^4^^,^^5^ (AAV8/P3-Alb-mNG). A few weeks after systemic administration (i.v. or i.p.) at an AAV dosage of $2\times 10^{11}\;\mathrm{vg}$ (viral genome) in adult mice, sufficient plasma fluorescence signals were obtained for two-photon imaging of cerebral cortical microcirculation. The expression level was sustained for at least a few months, allowing for longitudinal and repeated imaging of cortical microcirculation dynamics in mice. In the current study, we aimed at enhancing the utility of the promoter-driven plasma labeling AAV method in three ways: (1) by incorporating a brighter, more photostable green fluorescent protein, (2) by changing the administration route for a successful secondary AAV injection, and (3) by introducing an immunomodulation step to reinforce expression.

In our previous work, we chose mNeonGreen as a fluorescent tag for albumin because it was the brightest fluorescent protein^6^ then. In recent years, brighter green fluorescent proteins were reported, including StayGold^7^ and its monomeric variations StayGold(E138D)^8^ and mStayGold.^9^ Notably, StayGold-based fluorescent proteins feature high photostability (photobleach resistant), which is advantageous for continuous and/or high-magnification imaging. Here, we sought to evaluate these fluorescent proteins as a tag for albumin (Alb-SG, Alb-SG(E138D), and Alb-mSG, respectively) for imaging microcirculation in the mouse brain.

Next, we addressed the well-established challenge that neutralizing antibodies generated following AAV inoculation reduce the effectiveness of subsequent intravenous AAV injections.^10^^,^^11^ Remarkably, we were able to demonstrate additional Alb-mNeonGreen expression in the blood plasma when i.p. was chosen as an administration route. Furthermore, we demonstrate that boosting primary AAV injection is possible by co-injection of immunosuppressants, whereas our attempt to develop immune tolerance for the same AAV in neonatal mice was unsuccessful. Together, this work broadens the utility of AAVs encoding fluorescent albumin and complements our existing genetic strategies for robust blood plasma labeling.^3^^,^^12^

## 2 Methods

### 2.1 DNA Plasmids for AAV

The DNA plasmids for pAAV-P3-Alb-mNG and pAAV-P3-Alb-RM, respectively, encoding mouse albumin tagged with mNeonGreen (mNG) or Rosmarinus (RM), have been published by Wang et al.^2^ and are available from Addgene (plasmid ID: 183460 and 183462, respectively). For the synthesis of pAAV-P3-Alb-SG, we used Sequence Manipulation Suite^13^ to obtain a DNA sequence for the StayGold amino acid sequence.^7^ SG and SG(E138D) sequences were codon-optimized for the mouse using Twist Bioscience online software. As there is a single cutting site for NdeI in the Alb sequence of pAAV-P3-Alb-mNG, we synthesized a codon-optimized DNA sequence that includes NdeI, the partial albumin sequence, ScFV linker, and StayGold^7^ with a stop codon, NotI, and EcoRI (Twist BioScience, South San Francisco, California, United States). This DNA segment was ligated into pAAV-P3-Alb-mNG via NdeI and EcoRI. The monomeric variations of StayGold, SG(E138D),^8^ and mSG^9^ were artificially synthesized and incorporated into the pAAV-P3-Alb-* vector accordingly. AAVs were purified by the Gunma University Viral Vector Core or Viral Vector Facility VVF Zurich. pAAV-Alb-SG (213790) and pAAV-Alb-mSG (234549) are available from Addgene.^14^ pAAV-Alb-SG(E138D) is available upon request.

### 2.2 Mice and AAV Injection

**Fig. 1:**
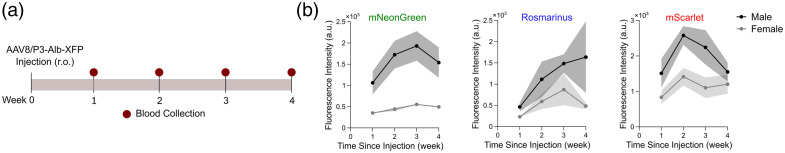
AAV-mediated plasma Alb-XFP expression is superior in male mice. (a) Experimental timeline showing retro-orbital (r.o.) injection of AAV8/P3-Alb-XFP and subsequent weekly blood collection. (b) Comparison of fluorescence intensity between male and female mice following systemic injection of AAV8/P3-Alb-XFP expressing mNeonGreen, Rosmarinus, and mScarlet (left to right panels) (n=2 to 6 mice per sex). Two-way ANOVA for sex: $F_{1,6}=3.721$, p=0.1020 for mNeonGreen; $F_{1,3}=3.301$, p=0.1669 for Rosmarinus; $F_{1,6}=7.231$, p=0.0361 for mScarlet. All graphs show means ± SEM.

Adult C57BL/6JRj mice of both sexes were used for experiments shown in Fig. 1, whereas the remaining experiments were conducted using both infant and adult male C57BL/6JRj. Mice were housed in a 12-h light/12-h dark cycle (lights on: 7 am) with food and water *ad libitum*. The procedures involving animal care, surgery, *in vivo* imaging, and sample preparation were approved by the local research ethics committee (Department of Experimental Medicine, University of Copenhagen) and conducted in accordance with the Danish Animal Experiments Inspectorate.

Expression of fluorescent protein-tagged albumin was achieved by systemic administration of AAV diluted in sterile phosphate-buffered saline (PBS). Intraperitoneal (i.p.) or intravenous injection via retro-orbital sinus (r.o.) was performed for adult mice. In neonatal mice, subcutaneous (s.c.) injection was preferred as it is the administration route with the highest injectable volume in early postnatal days^15^ and as it avoids causing damage to the vital milk sac compared with i.p.^16^ Viral loads of $2\times 10^{11}\;\mathrm{vg}$ and $5\times 10^{10}\;\mathrm{vg}$ were used for adult and neonatal injections, respectively. Mice were briefly anesthetized with isoflurane before AAV injection and were allowed to recover in a home cage thereafter under supervision.

### 2.3 *Ex Vivo* Macro Fluorescence Imaging

*Ex vivo* macro fluorescence imaging of blood samples was used to examine the development of fluorescently tagged albumin expression in the same animal over weeks as previously described.^2^^,^^3^ Briefly, a few microliters of blood samples were collected in glass capillaries, and fluorescence was examined under a macroscope (Leica M205 FA, Wetzlar, Germany). Filter sets ET GFP (excitation 470/40×, emission 525/50 m, 10447408, Leica), ET CFP (excitation 436/20×, emission 480/40  m, 10447409, Leica), and ET mCherry (excitation 560/40×, emission 630/75  m, 10450195, Leica) were used to image green, cyan, and red, respectively.

### 2.4 Analysis of *Ex Vivo* Macro Fluorescence Images

Because StayGold family’s excitation and emission spectra differ from Alb-mNG, a linear adjustment was made according to the spectral profiles documented on The Fluorescent Protein Database.^17^ Accordingly, mSG and SG(E138D) signals obtained through the ET GFP filter set (excitation 470/40×, emission 525/50  m, 10447408, Leica) were normalized to mNG by multiplying 0.84 and 0.81, respectively.

### 2.5 Immunosuppressant Administration

For experiments described in Sec. 3.5, the immunosuppressant cyclophosphamide (CP) was administered to adult mice in two schemes. High-dose scheme: 80  mg/kg CP was administered i.p. 4 h before and 3 days after AAV injection. Low-dose scheme: 10  mg/kg CP was administered i.p. 4 h before and 1, 2, and 3 days after AAV injection.

### 2.6 *In Vivo* Two-photon Imaging

The two-photon microscope setup consisted of a Bergamo microscope (Thorlabs, Newton, New Jersey, United States) equipped with a resonant scanner, a Chameleon Vision 2 laser (Coherent, Lubeck, Germany), an objective lens (XLPlan N; x25 NA = 1.05; Olympus, Hachioji, Tokyo, Japan), and the primary dichroic mirror FF705-Di-1-25x 36 (Chroma, Bellows Falls, Vermont, United States). Emission light was separated by the secondary dichroic mirror (FF562-Di03, Semrock, Rochester, New York, United States) with band-pass filters FF01-483/32-25 for RM and FF03-525/50 for mNG and mSG (both Semrock). Excitation wavelength was 850 nm for RM and 940 nm for mNG and mSG. The laser power was measured under the objective lens by a power meter (Thorlabs) before imaging to ensure consistency between imaging sessions. *In vivo* imaging was performed on anesthetized (70  mg/kg ketamine, 20  mg/kg xylazine) mice.

Cortical vasculature was imaged as described by Wang et al.^2^ Briefly, the mouse was subjected to a headplate implantation. A cranial window was made using a dental drill at a position corresponding to the somatosensory cortex, and the dura was surgically removed. The cranial window was sealed with a cover slip for microscopic observation. Ear skin imaging was performed by supporting the mouse in a lateral decubitus position and gently clamping the ear skin onto a glass slide.

### 2.7 Photobleaching of ECS Alb-XFP in *Ex Vivo* Ear Tissue

A small biopsy was obtained from the mouse ear (as in earmarking), immediately mounted on a glass slide with hydrogel/saline and sealed with a cover slip for microscopic observation, as previously described.^18^ The interstitial space of the ear dermis was observed continuously by two-photon microscopy at a frame rate of 1 Hz over an area of 50×50  μm (940 nm, 30 mW) for 5 min. For each recording, the mean fluorescence intensity change over time was measured and normalized to the mean intensity of the first three frames. Grouped mean normalized traces between 0 and 150 s were fitted to an exponential decay function $y=ae^{-bx}+c$ to estimate the decay constant b, using the curve fitting toolbox in MATLAB (MathWorks).

### 2.8 *In Vivo* Macroscopic Imaging

The same macroscope used for *ex vivo* macro fluorescence imaging (Sec. 2.3) was used for imaging of the cranial window in mice injected with AAV/P3-Alb-RM. Mice were anaesthetized (70  mg/kg ketamine, 20  mg/kg xylazine), head fixed to a MAG-1, and placed under the macroscope. Filter set ET CFP (excitation 436/20×, emission 480/40  m, 10447409, Leica) was used to image cyan.

### 2.9 Statistical Analysis

All values are indicated as mean ± SEM. Comparisons between multiple group means were assessed by either one-way or two-way ANOVA. Statistical significance was set to 5% (*P<0.05). Graph Prism 10 was used for all statistical analyses.

## 3 Results

AAVs for expression of fluorescent protein-tagged albumin (Alb-XFP) were produced by transfecting the pAAV-P3-XFP plasmid into HEK293-T along with helper plasmids. Green fluorescence signals after transfection were confirmed visually by fluorescence microscopy before AAVs were purified.

### 3.1 Superior Expression of Plasma Label in Male Mice

**Fig. 2:**
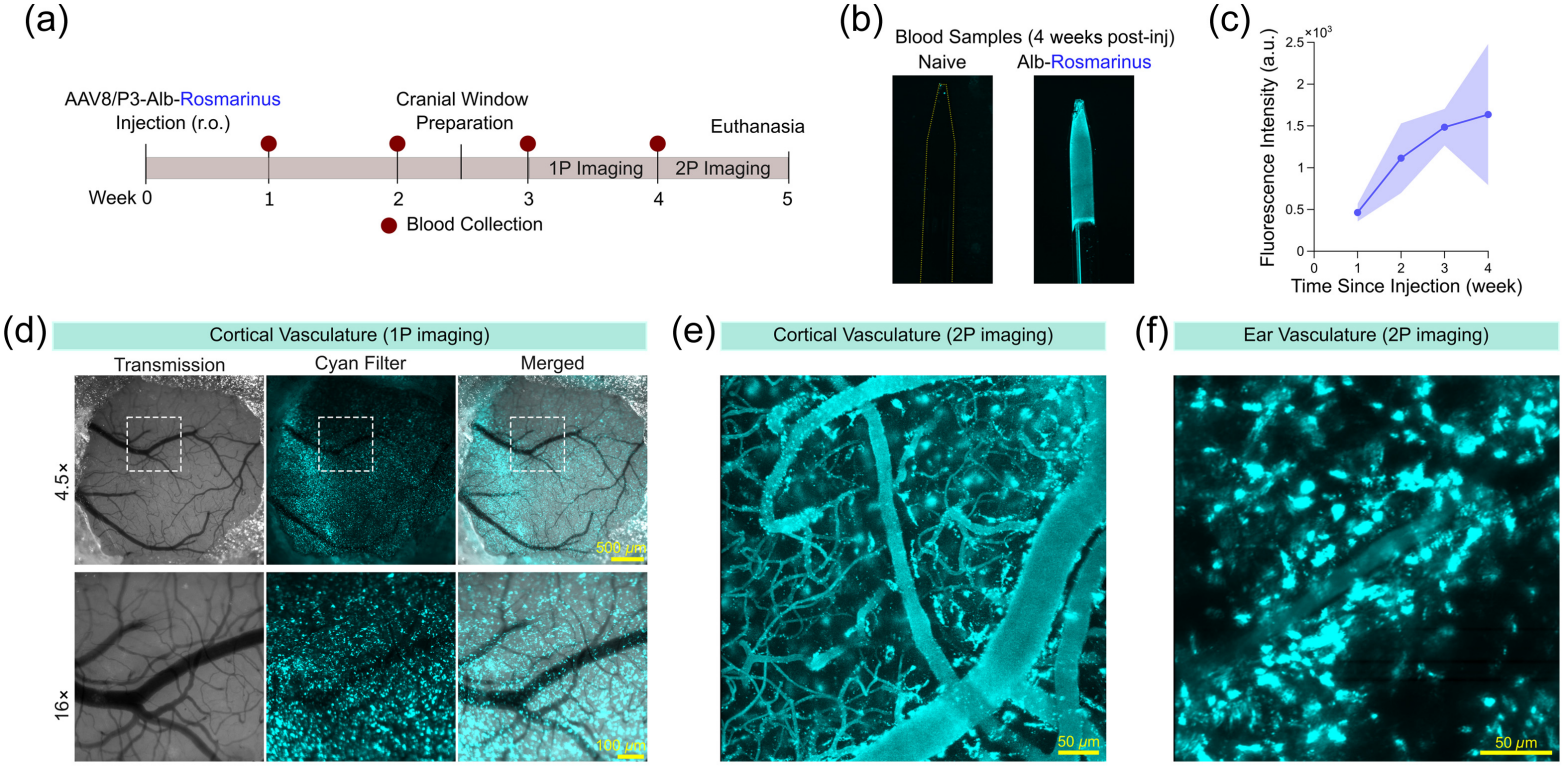
Profile of blood plasma labeling by AAV8/P3-Alb-mRM. (a) Experimental timeline. (b) Examples of blood sample fluorescence. (c) Blood Alb-RM signal intensity with respect to time since injection. (d) Macroscopic image of cranial surface vasculature. Numerous cyan fluorescent aggregates are observed on the brain surface. (e) Two-photon image of parenchymal vasculature in the cerebral cortex. Cyan fluorescent aggregates are present at the periphery of blood vessels. (f) Two-photon image of ear skin. Similar to the brain, intense cyan fluorescent aggregates are present.

Following previous studies reporting a higher infectability of AAV8 to hepatocytes in males than in females,^19^^,^^20^ we first compared the fluorescence signal intensity of plasma in mice injected with AAV8/P3-Alb-mNG, -RM, and -mSc by sex ($2\times 10^{11}\;\mathrm{vg}$, r.o.). Blood samples were collected weekly in glass capillaries and subjected to fluorescence intensity measurements [Figs. 1(a) and 2(a) for AAV8/P3-Alb-RM expression]. We found that male mice consistently yielded higher blood fluorescence signals than females throughout the examined period of four weeks [Fig. 1(b)]. Thus, we chose to use male mice for further experiments.

Of note, mice injected with our previously published AAV8/P3-Alb-RM^2^ exhibited robust cyan fluorescence but also developed aggregates in brain and ear tissue [Figs. 2(b)–2(f)] possibly due to RM’s tetrameric nature.

### 3.2 Alb-mNG Is Brighter but Less Photostable than Alb-mSG

**Fig. 3:**
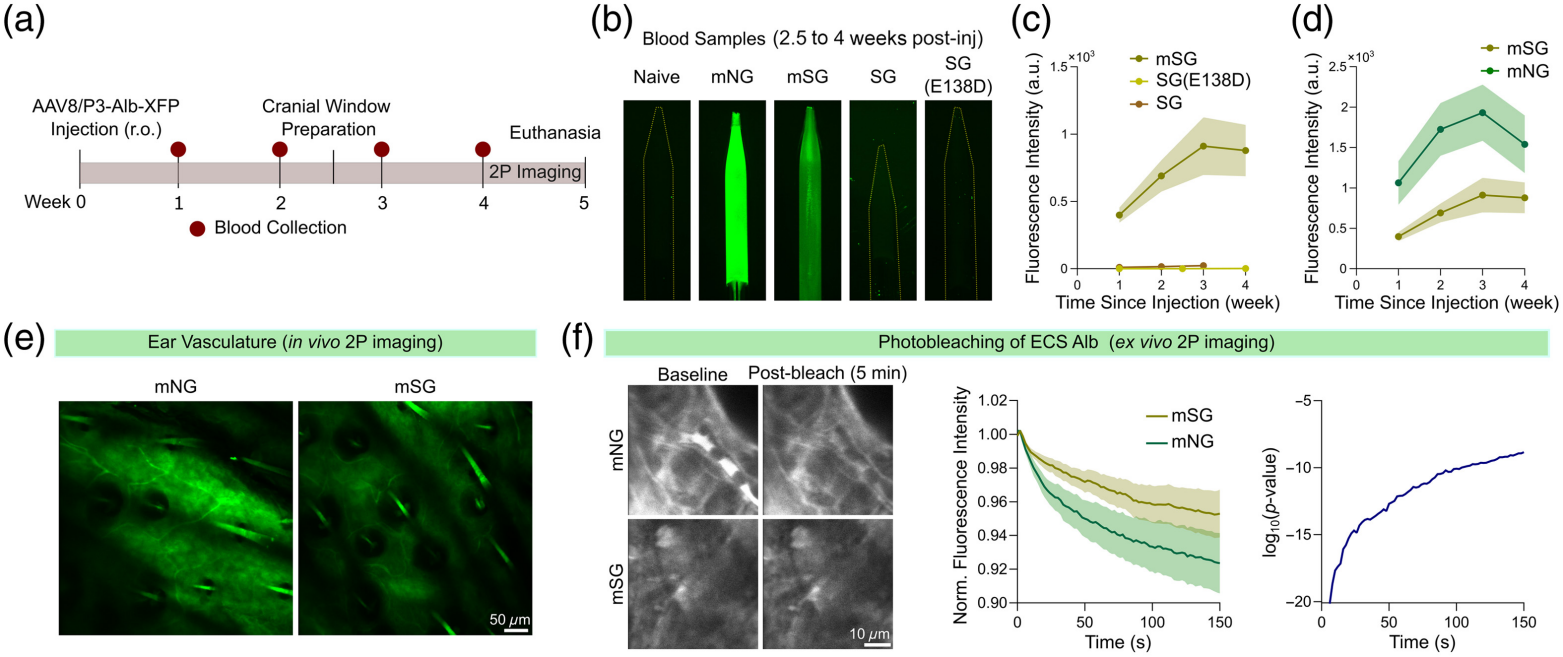
AAV-mediated plasma Alb-mNG is brighter but less photostable than Alb-mSG. (a) Experimental timeline showing retro-orbital (r.o.) injection of AAV8/P3-Alb-XFP and subsequent weekly blood collection. (b) Examples of fluorescent blood samples in glass pipettes were observed using a fluorescent microscope. (c) Comparison of fluorescence intensity of blood samples following systemic injection of AAV8/P3-Alb-mSG, -SG(E138D), and -SG (n=2 to 8 mice per group). (d) Comparison of fluorescence intensity of blood samples between AAV8/P3-Alb-mSG and AAV8/P3-Alb-mNG (n=6 to 8 mice per group). Two-way ANOVA for AAV type: $F_{1,12}=4.774$, p=0.0494. (e) *In vivo* two-photon imaging of mouse ear skin showing fluorescent albumin in capillaries and in the interstitial space. Hair shafts appear bright due to intrinsic autofluorescence. (f) Photostability assessment through continuous two-photon imaging of the interstitial space in mouse *ex vivo* ear tissue. Data are shown as mean ± shaded SEM (middle panel). P value assessment for comparing Alb-mSG vs. Alb-mNG. All graphs show means ± SEM (except P value assessment graph).

To introduce the highly photostable and bright green fluorescent protein StayGold to the genetically encoded, albumin-based plasma label, AAVs expressing Alb-SG, Alb-SG(E138D), or Alb-mSG were injected into adult male C57BL/6 mice [$2\times 10^{11}\;\mathrm{vg}$, r.o.; Fig. 3(a)]. Among the three constructs, Alb-SG and Alb-SG(E138D) exhibited rather little fluorescence intensity in the blood, despite visible expression in HEK293 culture (data not shown). By contrast, Alb-mSG displayed detectable signals that progressively became brighter during the first 3 weeks [Figs. 3(b) and 3(c)]. However, Alb-mSG showed lower signal intensity compared with Alb-mNG [Fig. 3(d)], albeit still detectable by *in vivo* two-photon microscopy in capillaries and interstitial fluid in the ear dermis [Fig. 3(e)]. Next, we tested the photostability of Alb-mNG and Alb-mSG by continuously imaging the albumin-containing interstitial fluid of *ex vivo* ear skin (see Sec. 2.7). Alb-mNG and Alb-mSG fluorescence signals decreased with time, but the decay was slower for Alb-mSG, indicating that Alb-mSG is more photostable than Alb-mNG [Fig. 3(f); decay constant: Alb-mSG $1.45\times 10^{-2}\;s^{-1}$; Alb-mNG, $2.10\times 10^{-2}\;s^{-1}$).

### 3.3 Secondary Intraperitoneal AAV Administration Results in Detectable Plasma Label

**Fig. 4:**
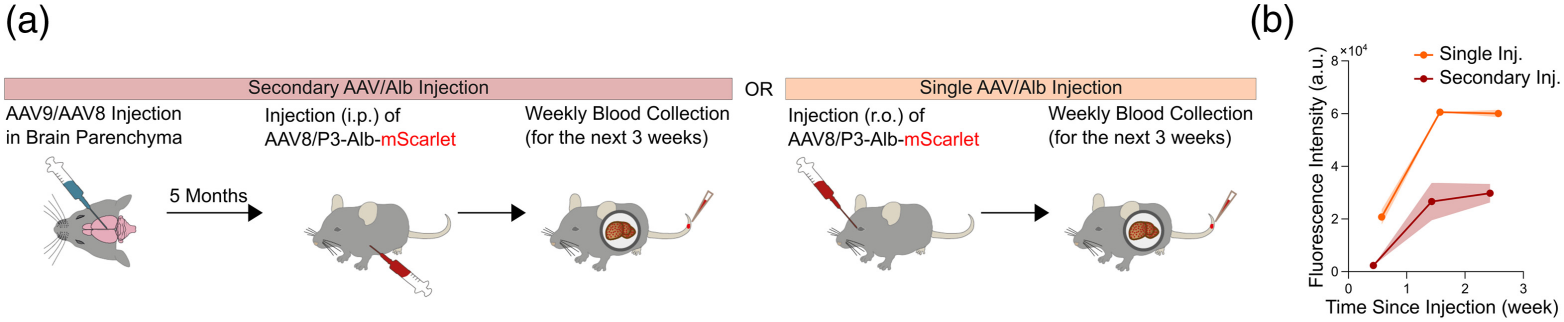
Secondary injection of liver-targeting AAV induces detectable levels of plasma Alb-mSc via intraperitoneal (i.p.) injection. (a) Experimental schedule illustrating the secondary AAV injection (purple) and single AAV injection schemes for AAV8-P3-Alb-mSc (n=3 to 5 mice per group). (b) Plasma mScarlet fluorescence comparing the single and secondary AAV injection schemes. Graph shows means ± SEM.

It is well established that once a mouse receives an AAV injection, even a local micro-injection into the brain, the following secondary AAV injection via the retro-orbital route days later will not be effective due to the activated immune system.^10^ We have confirmed this for AAV/P3-Alb-mSc when administered via the r.o. route in mice that had previously received a local AAV injection in the brain.^2^ Here, we tested the efficacy of AAV/P3-Alb-mSc by changing the re-administration route to the i.p. route in mice that received brain AAV microinjection 5 months earlier [Fig. 4(a)]. To our surprise, we were able to induce appreciable levels of plasma fluorescence expression, demonstrating the feasibility of i.p. injection as a viable route for AAV re-administration. Notably, the plasma label signal did not reach the same expression levels as a single retro-orbital injection in naïve mice [Fig. 4(b)].

### 3.4 Effects of Immunomodulation on Alb-mNG Expression

**Fig. 5:**
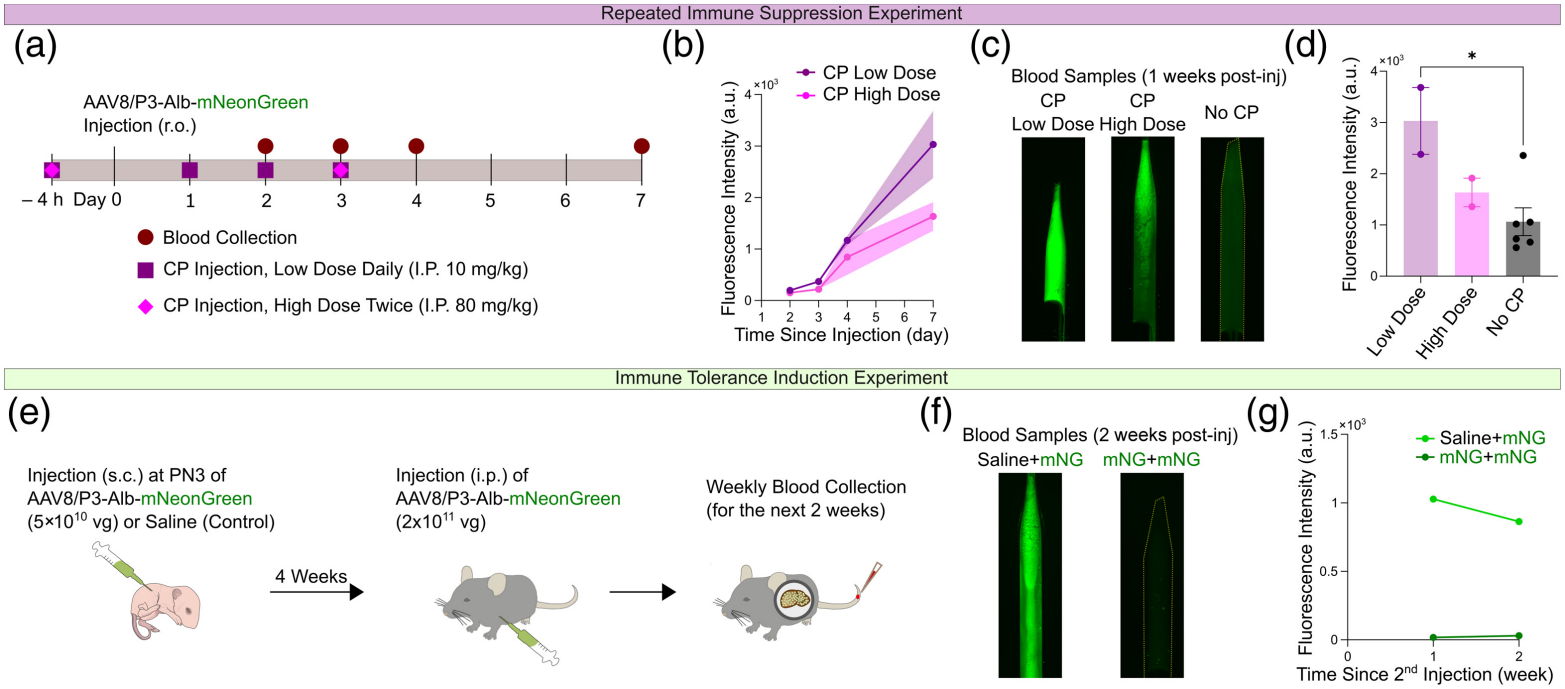
Immune suppression enhances AAV-induced plasma label expression. (a) Experimental timeline indicating CP and AAV injection timing and blood sample collection timing. (b) Plasma Alb-mNG signal intensity increases steadily under the two CP treatment schemes. (c) Examples of Alb-mNG fluorescence in blood collected on day 7. (d) Quantifications of plasma Alb-mNG signals after AAV injection on day 7. One-way ANOVA (multiple comparisons): high dose versus no CP, no significance; low dose versus no CP, P<0.05; $n_{\text{Low Dose}}=2$, $n_{\text{High Dose}}=2$, $n_{\text{No }\mathrm{CP}}=6$. (e) Experimental timeline of the immune tolerance experiment. (f) Examples of Alb-mNG fluorescence in blood collected on day 7. (g) PN3-inoculated mice (dark green, n=3 mice) did not express detectable plasma Alb-mNG signals, compared with a representative example of uninoculated single AAV injection (light green). All graphs show means ± SEM.

Although the main cause of suppressed infectivity after a second intravenous AAV injection is thought to be neutralizing antibodies against the AAV capsid,^10^^,^^11^ it is possible that the expressed recombinant X fluorescent protein (XFP) may also be recognized as nonself by the immune system and targeted for clearance. We tested this possibility by administering the immunosuppressant CP to mice before and in the early phase of AAV8/P3-Alb-mNG viral transduction, assuming that liver infection occurs predominantly within a day.^21^ CP preferentially ablates proliferating B and T cells, thereby reducing antibody production and nonself protein clearance.^22^ Two CP injection schemes were tested [Fig. 5(a)]: either a low dose (i.p. 10  mg/kg) administered daily from 4 h prior to AAV injection until 3 days after or a high dose (i.p. 60  mg/kg) administered only 4 h prior to and on the third day postinjection. We found that CP-treated mice generally increased plasma mNG fluorescence on day 7 after AAV injection [Figs. 5(b)–5(d)], suggesting that the immune system clears the recombinant Alb-mNG protein from circulation. In addition, we attempted to induce immune tolerance to AAV and/or Alb-mNG by inoculating AAV ($5\times 10^{10}\;\mathrm{vg}$) into postnatal day 3 (PN3) neonates so that these components could be recognized as self by the mice in the subsequent days. However, this strategy failed in developing immune tolerance as PN3-inoculated mice showed little detectable plasma Alb-mNG expression 1 to 2 weeks after a secondary AAV injection [Figs. 5(e)–5(g)].

## 4 Discussion

### 4.1 Sex Differences of AAV-Mediated Plasma Labeling in Mice

Numerous studies have demonstrated that hepatocytes in male mice are transduced more efficiently by AAV8 than those in females.^19^^,^^20^^,^^23^ Consistent with these reports, our AAV8-mediated fluorescent plasma labeling approach revealed a similar sex bias, with blood fluorescence levels remaining consistently higher in males compared with females 4 weeks after viral injection [Fig. 1(b)]. This difference was independent of the specific fluorescent protein fused to albumin.

The biological mechanisms underlying this sex bias are not yet fully resolved. AAV transduction begins with vector binding to cell-surface glycans and receptors, with glycosylation and expression patterns that may differ between sexes. However, several studies have reported comparable hepatic AAV genome copy numbers in males and females,^19^^,^^23^ suggesting that the bias arises downstream—at the level of transgene expression rather than viral uptake.

Supporting this idea, Davidoff et al.^23^ demonstrated that androgens play a critical role in AAV-mediated hepatic transduction in mice. Androgens influence the binding of nuclear regulatory proteins to the viral genome, thereby enhancing transgene transcription. Castration of male mice prior to AAV administration reduces transduction efficiency to levels observed in females, whereas oophorectomy in females has little effect. Conversely, treatment of oophorectomized females with the androgen 5α-dihydrotestosterone prior to AAV injection restores hepatic transduction and transgene expression to male-like levels.

An immediate solution for effective expression of Alb-XFP in female animals is increasing the AAV8 dosage (even 2 to 3×). Alternatively, other AAVs with liver tropism, such as AAV9, could be tested for any sex-dependent expression.

Together, these reported findings underscore the importance of considering sex as a biological variable in AAV-based liver-targeted gene delivery. Given the growing emphasis on sex inclusion in biomedical research and the potential translational implications of these sex-dependent differences in hepatic transduction, further investigation into the molecular and hormonal mechanisms driving this bias is warranted.

### 4.2 StayGold Variants as a C-terminal Tag for Albumin

In the current work, we sought to improve the utility of AAV-mediated blood plasma label^2^ by producing new variants of Alb-XFP. First, we introduced the highly bright and photostable green fluorescent protein StayGold. To our disappointment, we were unable to detect visible Alb-StayGold signals [Fig. 3(c)]. We speculate that the dimer-forming property of StayGold might have led to the absence of a fluorescence signal; hence, we tested StayGold(E138D), which was published for its monomer-forming property. Nonetheless, the plasma signal intensity of Alb-SG(E138D) was impractically low for imaging purposes. By contrast, Alb-mSG was detectable in blood plasma. These results are in part in line with a recent paper on mBaoJin,^24^ another monomeric StayGold variant, which reported superior performance of mSG and mBaoJin to SG, SG(E138D), and mNG when expressed in neurons in the mouse cerebral cortex.

We report that Alb-mNG results in significantly brighter plasma signals than Alb-mSG. Multiple factors may have contributed to the unanticipated underperformance of StayGold and its variants when used as a C-terminal tag of albumin. First, StayGold variants appear to be sensitive to N-terminal residues.^25^ As the fluorescent protein is linked via the GGGGS × 3 scFv linker in Alb-XFP,^2^ it is possible that the flexible linker or the conformation of the fused albumin compromised the fluorophore of the StayGold variants. With the advent of algorithm- or machine learning-based prediction of fluorescent protein brightness,^26^^,^^27^ future approaches should include *in silico* evaluation of recombinant plasma probes. Second, Viola et al.^28^ observed that SG(E138D) is ∼20% dimmer than mSG when expressed on an I3-01 nanocage, whereas the SG(E138D) fluorescence signal per transfected cultured human retinal pigment epithelial cell was substantially lower (<70%). The authors noted that a possible transcription inefficiency of their codon-unoptimized SG(E138D) sequence may have contributed to the dimness. Indeed, the mSG sequence in Alb-mSG (i.e., Addgene 212019 seq 419223) had a highly optimized codon adaptation index (CAI, calculated using GenScript OptimumGene Codon Optimization Tool) of 0.98, whereas CAI of the SG and mSG(E138D) sequences were both 0.76. Hence, there is room for further codon optimization for SG and mSG. Finally, considering that both Alb-SG and Alb-SG(E138D) were visible following HEK293 transfection, it is possible that postsecretory modifications in blood compromised the fluorescent proteins. Although not published, we were unable to detect blood fluorescence signals from Alb-mNeptune2.5 and Alb-SuperNova in our experiments. By contrast, the tetrameric Alb-RM yielded bright blood labeling but at the cost of aggregate formation (Fig. 2). Identifying common features among these suboptimal fluorescent proteins used as a C-terminal albumin tag may provide insights into designing a more effective plasma label.

Despite the relative dimness of plasma Alb-mSG as expressed via hepatocytes, we confirm that Alb-mSG is more resistant to photobleaching than Alb-mNG [Figs. 3(d) and 3(f)]. As albumin infiltrates the interstitial space in peripheral tissues, Alb-XFP could be used for shadow imaging^12^ (inverted imaging of cellular structure by imaging the fluorescence-labeled extracellular space^29^^–^^31^). The photostable Alb-mSG thus provides a strong advantage for time-lapse imaging for (extra)cellular morphology over Alb-mNG. In addition, although Alb-mNG remains the optimal choice for most blood vessel studies, Alb-mSG is highly suitable for imaging of albumin-containing fluids with slower flow dynamics, such as lymph or interstitial fluid. Furthermore, we expect that Alb-mSG will be desired in multiphoton imaging for deep tissue penetration or super-resolution enhancement, whereby excitation light intensity becomes relatively high.^32^^,^^33^ We find that Alb-mSG’s resistance to photobleaching, compared with Alb-mNG, was less prominent than previously reported.^7^^,^^9^ This could possibly be due to the diffusion of Alb-XFP into the bleached area. Because the diffraction-limited focal spot of two-photon excitation is a few micrometers in the axial direction, intact Alb-XFP molecules could easily diffuse into the photobleached plane from above or below. Likewise, photobleached Alb-XFP molecules can diffuse out.

### 4.3 Alb-XFP Expression and the Immune System

It is well established that secondary systemic AAV injections do not yield expression due to the immune response primed during the first AAV administration.^10^^,^^11^ We serendipitously found that practically imageable plasma fluorescence is achieved if a secondary injection of AAV/P3-Alb-XFP is made via the intraperitoneal route (Fig. 4), whereas it fails if made via the retro-orbital route.^2^ This allows long-term plasma fluorescence expression in mice that have previously received AAV in the brain or elsewhere, thereby extending the utility of the current AAV-based plasma labeling strategy.

Our speculation is that intravenous AAV injection into the tail vein or retro-orbital AAV injection infects the liver via the hepatic artery and portal vein, with presumably numerous rounds of systemic circulations. The extended exposure in the bloodstream likely increases the chance of AAV being neutralized by circulating antibodies. By contrast, the intraperitoneal route allows gradual viral entry into the circulation via the blood vessels associated with the intestines and other digestive organs, directing AAV to the liver primarily via the portal vein with shorter exposure to blood.

Neutralizing antibody generation takes several days, whereas AAV particles typically remain in the bloodstream for less than a few hours.^21^ We successfully enhanced Alb-XFP expression by quasi-co-injection of AAV and the immune suppressor CP [Figs. 5(a)–5(d)]. Interestingly, the low-dose scheme seems more effective in boosting expression compared with the high-dose scheme, highlighting the highest importance of prolonged treatment over high dosage. Thus, CP seems to both improve AAV transduction efficiency by reducing immune clearance of AAV particles and increase the half-life of plasma Alb-XFP by reducing its active clearance by the immune system. Further testing is required to identify the major contributor. Nonetheless, an optimized immune tolerance strategy should consider both the AAV capsid and the secretory protein. The combination of AAV and CP could enable longitudinal imaging of the vascular development in the neonate mouse brain as dynamic angiogenic processes during the developmental period have been described recently.^34^

Finally, we attempted to circumvent the immune system by establishing immune tolerance, taking advantage of the fact that neonatal mice are immunologically less mature at birth than human newborns and have a postnatal window of several days to recognize nonimmunogenic self-proteins.^35^ In addition, virus-mediated immune tolerance has been reported to depend on dosage.^36^^,^^37^ Although our attempt with $5\times 10^{10}\;\mathrm{vg}$ to PN3 neonates was not successful [Figs. 5(e)–5(g)], a previous study succeeded in establishing immune tolerance of transgene-expressed protein using AAV8 at a dosage of 3 to $5\times 10^{9}\;\mathrm{vg}$ in PN2 BALB/c neonate mice (assuming 2 to 3 g bodyweight).^37^ The immune tolerance reported by this study was achieved when the primary administration occurred at PN1–2 and to a lesser extent at PN7, indicating a critical early window for induction of immune tolerance. Our PN3 injection falls within this apparent window, though an earlier time point might be more optimal. One factor potentially contributing to the failed induction of immune tolerance could be the significantly higher AAV amount. Although neonatal AAV delivery induces immune tolerance to transgene products, tolerance to the AAV capsid itself has not yet been achieved.^37^^–^^39^ Accordingly, primary administration in PN1–2 neonates has been reported not to elicit detectable levels of anti-AAV antibody production.^39^ By contrast, a secondary injection later in life triggers a robust antibody response, alluding to the possibility that the transient surge of AAV capsid exposure (rather than the relatively gradual transgene expression) establishes immune memory by some mechanism.

## 5 Conclusion

In this study, we improved our albumin-based plasma labeling method by: (1) introducing Alb-mSG as a more photostable, albeit less bright, alternative to Alb-mNG; (2) enabling successful secondary AAV injection via the intraperitoneal route; and (3) enhancing Alb-mNG expression through co-treatment with immunosuppressants. Although further improvements, such as induction of immune tolerance, remain possible, these optimizations have broadened the utility and applicability of Alb-XFP as a plasma marker for studying blood and other albumin-containing fluids.

## Data Availability

DNA plasmids and their sequences are available from Addgene.org.
